# Red blood cell dynamics in extravascular biological tissues modelled as canonical disordered porous media

**DOI:** 10.1098/rsfs.2022.0037

**Published:** 2022-10-14

**Authors:** Qi Zhou, Kerstin Schirrmann, Eleanor Doman, Qi Chen, Naval Singh, P. Ravi Selvaganapathy, Miguel O. Bernabeu, Oliver E. Jensen, Anne Juel, Igor L. Chernyavsky, Timm Krüger

**Affiliations:** ^1^ School of Engineering, Institute for Multiscale Thermofluids, Edinburgh, UK; ^2^ Centre for Medical Informatics, The University of Edinburgh, Edinburgh, UK; ^3^ The Bayes Centre, The University of Edinburgh, Edinburgh, UK; ^4^ Manchester Centre for Nonlinear Dynamics, Manchester, UK; ^5^ Department of Physics and Astronomy, The University of Manchester, Manchester, UK; ^6^ Department of Mathematics, The University of Manchester, Manchester, UK; ^7^ Maternal and Fetal Health Research Centre, School of Medical Sciences, The University of Manchester, Manchester, UK; ^8^ Department of Mechanical Engineering, School of Biomedical Engineering, McMaster University, Hamilton, Canada

**Keywords:** haemodynamics, red blood cells, biological tissues, porous media, lattice-Boltzmann method, microfluidics

## Abstract

The dynamics of blood flow in the smallest vessels and passages of the human body, where the cellular character of blood becomes prominent, plays a dominant role in the transport and exchange of solutes. Recent studies have revealed that the microhaemodynamics of a vascular network is underpinned by its interconnected structure, and certain structural alterations such as capillary dilation and blockage can substantially change blood flow patterns. However, for extravascular media with disordered microstructure (e.g. the porous intervillous space in the placenta), it remains unclear how the medium’s structure affects the haemodynamics. Here, we simulate cellular blood flow in simple models of canonical porous media representative of extravascular biological tissue, with corroborative microfluidic experiments performed for validation purposes. For the media considered here, we observe three main effects: first, the relative apparent viscosity of blood increases with the structural disorder of the medium; second, the presence of red blood cells (RBCs) dynamically alters the flow distribution in the medium; third, symmetry breaking introduced by moderate structural disorder can promote more homogeneous distribution of RBCs. Our findings contribute to a better understanding of the cell-scale haemodynamics that mediates the relationship linking the function of certain biological tissues to their microstructure.

## 1. Introduction

Significant progress in computational modelling has been made over recent years to elucidate the complex behaviour of blood flow in physiological environments, e.g. the small-vessel network in the brain [1–3], in the eye [4,5], in tumours [6] and in microaneurysms [7]. However, the flow and transport of blood and solutes in other (e.g. extravascular) types of biological media with vital function, such as the intervillous space (IVS) of the human placenta featuring a highly disordered network of pores and flow passages of size comparable to that of red blood cells (RBCs), remains poorly understood [8,9].

Comprehensive theories of flow and transport in porous media have been established, revealing subtle relationships between pore-scale structural heterogeneity and macroscopic flow properties [10–13]. However, existing models of flow through porous media often assume a homogeneous fluid and cannot accurately infer the intricate blood rheology in living biological media [14], e.g. the IVS, where the particulate and highly confined character of blood plays a key role, introducing spatio-temporal variability and nonlinearity beyond the description of prevalent continuum models [15,16].

Emerging cell-resolved models of blood flow using advanced mesoscopic methods [4,17–20] have been extensively applied since the late 2010s to simulate multiscale haemodynamics in synthetic or realistic vascular networks. These simulations have greatly improved our understanding of microscopic processes in the blood stream mediated by the flowing RBCs within, e.g. biased haematocrit distribution and oxygen transport arising from abnormal branching patterns of the vasculature [21].

Facilitated by robust image segmentation and meshing techniques [22,23], cell-resolved models are now technically applicable to living porous media. These models provide a promising avenue for microscopic characterization of the microstructure-dependent cellular blood flow, which can reproduce haemorheological behaviour based on first principles and inform generalized constitutive relationships needed by more robust continuum models [15,24]. A key aim is the development of effective reduced-order models for efficient simulation of large tissue/organ systems [25]. Additionally, cellular simulations can help design and optimize microfluidic oxygenators that serve as artificial lung assist devices [26].

In this work, we aim to characterize microscopic blood flow in canonical porous medium models constructed to represent simplistic extravascular biological media, for which we have control over the structural characteristics that can be related to physiological or pathological conditions of porous tissues and organs. Specifically, our focus is to quantify the correlations between cell-scale haemodynamics (e.g. flow patterns, RBC partitioning, haematocrit distribution) and key metrics of the porous medium, such as porosity and disorder. Primarily, we tackle the task computationally, with the aid of microfluidic experiments in equivalent flow systems for validation.

## 2. Methods

### 2.1. Design of canonical disordered porous media

**Figure 1.**
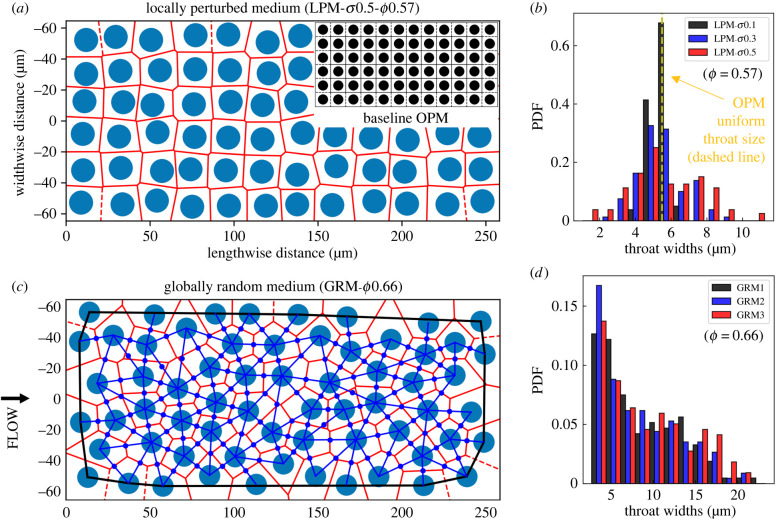
Construction of canonical porous media. (*a*) Locally perturbed medium (LPM), constructed by introducing local perturbations to a periodic-ordered medium (OPM) of square obstacle arrays (inset). The throats (red edges) are delineated by Voronoi tessellation (red polygons); the pores are located where neighbouring edges meet. (*b*) Throat-width distributions (in the form of normalized histograms) for LPM geometries perturbed from an OPM of porosity *ϕ* = 0.57, with incremental disorder *σ* = 0.1, 0.3, 0.5. (*c*) Globally random medium (GRM) with pores and throats (excluding those in direct contact with the boundaries, which are located outside the black bounding box) delineated by Voronoi tessellation (red polygons) and Delaunay triangulation (blue triangles). The blue dots indicate the middle points of pore openings where local flows and cell fluxes are evaluated. (*d*) Throat-width distributions for three GRM realizations (*ϕ* = 0.66). For both LPM and GRM, flow is driven in the horizontal direction.

We consider two typical designs of planar (quasi-two-dimensional) canonical disordered porous media (DPM) for our three-dimensional simulations and validating experiments: *locally perturbed media* (LPM) (figure 1*a*) representing weak-disorder systems and *globally random media* (GRM) (figure 1*c*) representing strong-disorder systems, both of which are in the form of non-overlapping uniformly sized cylinders. The design process is inspired by the ‘bundle of tubes (uniform)’ model implemented by Gostick *et al.* [27].

To obtain LPM, we first construct an ordered porous medium (OPM) by placing cylinders of constant diameter *D*_*c*_ (determined by porosity *ϕ* and domain size) on a square grid with length *L* and width *W* (figure 1*a* inset), which can be regarded as a generalized capillary bundle model. Three porosity values *ϕ* = 0.57, 0.62, 0.67 are considered to account for distinct levels of confinement that cellular blood flow experiences in the capillary bed and extravascular tissues, corresponding to a confinement ratio of *χ* = 1.2, 1.0, 0.9 (defined as *χ* = *D*_RBC_/*W*_throat_, which compares the effective RBC diameter with the size of pore-throats) in the baseline OPM geometry, respectively. The alteration of porosity is achieved by varying *D*_*c*_ while keeping the cylinder positions unchanged. The structural disorder of the LPM is then created by introducing random perturbations to the cylinder positions (*x*, *y*) with standard deviation *σ* = 0.1, 0.3, 0.5, 0.7, where (*x*, *y*) are drawn from a bivariate normal distribution. For a relatively large *σ* (e.g. *σ* = 0.7), the perturbations are likely to cause overlapping cylinders and several attempts may be needed to construct a non-overlapping layout. The resulting distributions of throat widths in the LPM are Gaussian (figure 1*b*).

The GRM is constructed by placing a uniformly random spatial distribution of cylinders in a domain of given size for a designated porosity (*ϕ* = 0.66), while enforcing a minimal separation distance of 0.4*D*_RBC_ between neighbouring cylinders (figure 1*c*). The resulting distribution of throat widths in the GRM is approximately exponential (figure 1*d*). Three GRM realizations are tested in the present study.

For ease of description when comparing various geometries in plots, each LPM geometry is assigned with a disorder identifier and a porosity identifier, e.g. LPM-*σ*0.5-*ϕ*0.57 for LPM with *σ* = 0.5, *ϕ* = 0.57. For OPM and GRM, only a porosity identifier is assigned, e.g. OPM-*ϕ*0.57 and GRM-*ϕ*0.66.

### 2.2. Numerical model

The immersed-boundary lattice-Boltzmann method (IB-LBM [28,29]) simulates cellular blood flow as a suspension of deformable RBCs (see the electronic supplementary material, section S1 for more details of the model) through a shallow Hele–Shaw bed (*L* × *W* × *H* = 258 × 129 × 6 μm^3^) of vertical cylinders, which is constructed through extrusion over a distance (*H*) of the desired OPM or DPM (LPM and GRM) designs of dimension *L* × *W* in the depth direction. Each RBC is modelled using our previous approach [30,31] as a closed hyperelastic membrane with the unstressed shape of a biconcave discoid, which has been calibrated and benchmarked against experimental measurements of healthy human RBC [32]. The morphological deformation of RBCs in confined shear flows is primarily governed by the shear and bending elasticity of the RBC membrane, which contributes to characteristic features at the cell’s front and rear ends (Fig. S1 in the electronic supplementary material). To tackle close cell–cell and cell–wall interactions, a repulsion potential decaying with inverse distance between neighbouring surfaces is numerically implemented with interaction intensities comparable to the bending elasticity of the RBC membrane [31]. For a list of key simulation parameters, refer to the electronic supplementary material, table TS1.

**Figure 2.**
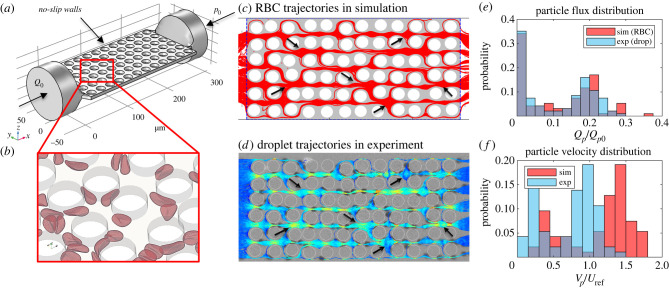
Cross-validation of simulation and experiment (LPM-*σ*0.5-*ϕ*0.57). (*a*) Schematic of the simulation domain and boundary conditions. (*b*) A snapshot of the simulated RBCs in the porous medium domain. (*c*,*d*) Particle trajectories over time. The red lines in (*c*) represent the simulated RBC trajectories, and the red dots indicate occluded throats by the RBCs. The coloured lines in (*d*) represent the droplet trajectories in experiment with high velocities marked in brighter colours. The black arrows in (*c*,*d*) highlight example regions of interest where simulation and experiment agree well. Comparison of (*e*) normalized particle flux distributions and (*f*) normalized particle velocity distributions between the simulation and experiment. See appendix A for the definition of symbols in (*e*,*f*).

In the simulations, an RBC-free plasma flow (Newtonian) is initialized from left to right along the channel axis (length direction) with a designated volumetric flow rate *Q*_0_ (figure 2*a*). The flow is driven by imposing a parabolic velocity profile at the inlet (assuming Poiseuille flow under *Q*_0_) and a reference pressure at the outlet (*p*_0_), through two cylindrical flow extensions smoothly stitched to the porous bed of rectangular cross-section (*W* × *H*). The no-slip condition is imposed at the wall and all fluid–solid interfaces. Once the plasma flow is converged, RBCs are randomly inserted from the inlet flow extension in a continuous manner with designated feeding discharge haematocrit (i.e. flow-weighted RBC volume fraction), *H*_*F*_ = 0.1, 0.2, 0.3. RBCs reaching the outlet flow extension are removed from the system (see electronic supplementary material, movie S1 for an example simulation in the OPM-*ϕ*0.57 geometry). For the definition of other flow-related quantities, refer to appendix A.

### 2.3. Microfluidic experiments

In the microfluidic experiments, a suspension of silicone oil droplets (Sigma Aldrich, viscosity *ν* = 20 cSt with paraffin oil dye) in a mixture of water and glycerol (Sigma Aldrich, 80 : 20 by volume with 0.2% SDS, viscosity 0.24 Pa s at $21^{\circ }C$) was used as biomimetic model for blood. The droplets were generated in a flow-focusing device [33] which was connected to the porous medium via a bent glass capillary (length 100 mm, outer diameter 1 mm, inner diameter 0.58 mm). Droplets with a diameter of 240 μm were produced by flowing the inner phase (silicone oil) at 4 μl min^−1^ and introducing the outer phase (water/glycerol) through a cross-junction at 5 μl min^−1^ immediately upstream of the flow-focusing channel constriction. Downstream of the droplet formation, another 11 μl min^−1^ of water–glycerol mix was added to set the droplet flow fraction to *H*_*F*_ = 0.2. Flow control at the different inlets was achieved with a combination of a pressure controller (Elvesys) fitted with flow meters and a syringe pump (KD Scientific). Both the flow-focusing device and the porous medium were made from PDMS (polydimethylsiloxane, Sylgard 184, Dow Corning). To obtain hydrophilic wetting behaviour, the devices were oxidized by oxygen plasma treatment (Henniker plasma HPT-100) and immediately filled with water. The porous medium corresponds to the LPM-*σ*0.5-*ϕ*0.57 geometry (i.e. disorder 0.5 and porosity 0.57) used in numerical simulations, scaled by the ratio of the droplet diameter to the equivalent diameter of simulated RBCs (*D*_RBC_ = 6.68 μm). The suspension flow was imaged with a monochrome CMOS camera (PCO 1200hs) and analysed off-line using the ImageJ platform TrackMate [34].

## 3. Results and discussion

We first cross-validate the simulated RBC dynamics in the porous media with a generic model of soft particle suspension flow using microfluidic droplets, aimed at reproducing the physical behaviour of cellular blood flow. Then, we demonstrate the effect of porous medium structure and the presence of RBCs on the flow resistance. Lastly, we investigate the RBCs’ contribution to pore-scale flow redistribution and their spatio-temporal dynamics in relation to structural characteristics of the porous domain.

### 3.1. Cross-validation of simulation and experiment

Good agreement between the simulated RBC dynamics and experimented droplet dynamics is obtained (see figure 2*c*–*f* and electronic supplementary material, movies S2 and S3 for comparison in an example geometry), including the pattern of particle trajectories (figure 2*c*,*d*), the distribution of particle fluxes (figure 2*e*) and the distribution of particle velocities (time-average speed for particles crossing individual pore-throats, figure 2*f*). Both the RBCs and droplets demonstrate evident preferential pathways or regional shunts when travelling across the porous domain, with discrete clusters of high-flux and low-flux throats. Similarly, the mean particle velocities at individual throats feature a bimodal distribution where high-velocity and low-velocity populations coexist.

Some quantitative discrepancies, e.g. in the magnitude of pore-scale velocities (figure 2*f*), stem from subtle differences between the two particle types [35]. First, the unstressed shape of a droplet is spherical, whereas that of the RBC is biconcave (suggesting a large excess area compared to an equivalent sphere of the same volume). Second, the intrinsic surface properties of a droplet and an RBC differ, the former of which relies on surface tension to resist deformation in shear flow, whereas the latter is primarily subject to membrane elasticity governing shear and bending stresses under low–moderate flow velocities [36] (as in the case of viscous flow through disordered porous media).

The encouraging agreement observed in the overall behaviour of particle trajectories, fluxes, velocities between the simulated RBCs and microfluidic droplets underpins the existence of potential physical determinants of the cell-scale haemodynamics in confined pore-space networks, e.g. the channel confinement (particle size relative to throat size) and the particle deformability (deforming stress relative to restoring stress). Such physical determinants can be universal regardless of certain particle properties such as size, shape or surface composition, therefore dominating the particulate flow behaviour in canonical porous media. Further characterization and understanding of these determinants can contribute to the development of robust blood analogues [37] that may serve as model systems of real blood with high-level rheological and haemodynamic similarity. Emerging biomimetic counterparts of RBCs include droplets, vesicles and capsules given their stable properties and versatile fabrication methods [37,38], which can reproduce key features of RBCs in shear flow and are envisioned for mimicking particulate blood flow in health and disease under well-controlled conditions.

### 3.2. Interplay of the porous structure and RBC presence on flow resistance and blood viscosity

To assess the resistance to plasma flow (RBC-free) and suspension flow (RBC-laden, with default feeding haematocrit *H*_*F*_ = 0.2), we compare the overall pressure drop Δ*P* across different porous media under an identical feeding volumetric flow rate at the inlet (*Q*_0_ = 0.4 μl min^−1^ in simulations, unless otherwise specified). For suspension flow, we also examine the relative apparent viscosity *η*_rel_ comparing the suspension apparent viscosity *η*_app_ to that of plasma *η*_0_ (1 × 10^−3^ Pa s), which can be calculated as the ratio of pressure drops for equivalent RBC suspension and Newtonian plasma flows under same inflow (*Q*_*S*_ = *Q*_*N*_ = *Q*_0_), i.e. *η*_rel_ = *η*_app_/*η*_0_ = Δ*P*_S_/Δ*P*_*N*_.

**Figure 3.**
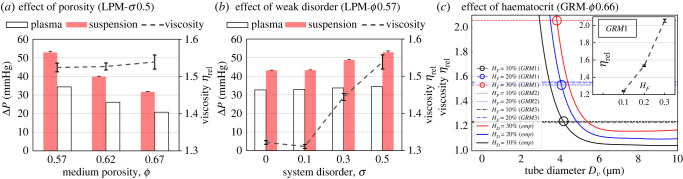
Evaluation of flow resistance in porous media with different geometries and flow conditions. Pressure drop across the simulated porous media under designated volumetric flow rates *Q*_0_ fed at the inlet (*Q*_0_ = 0.4 μl min^−1^ unless specified otherwise). The default porosity is *ϕ* = 0.57. (*a*) Variation of pressure drop against increasing porosity (*ϕ* = 0.57, 0.62, 0.67) in LPMs of fixed disorder *σ* = 0.5 for plasma flow and a suspension flow with RBCs of feeding haematocrit *H*_*F*_ = 0.2. The relative apparent viscosity *η*_rel_ of the suspension flowing within is assessed through comparing with plasma viscosity. The error bars represent standard deviation of measurements from five time instants of the suspension flow. (*b*) Variation of pressure drop against disorder (*σ* = 0.1, 0.3, 0.5) in LPMs of fixed porosity *ϕ* = 0.57 for plasma flow and a suspension flow with *H*_*F*_ = 0.2, where *η*_rel_ is quantified the same way as in (*a*). (*c*) Comparison of *η*_rel_ in three GRMs of fixed porosity *ϕ* = 0.66 (*GRM1, GRM2, GRM3*, horizontal lines) with empirical predictions for straight tubes *in vitro* at designated discharge haematocrits [39] (*emp*, solid curved lines). The round symbols indicate the characteristic diameter *D*_*v*_ of an equivalent tube where the suspension has the same relative apparent viscosity. The inset of (*c*) shows the variation of simulated *η*_rel_ against increasing *H*_*F*_ = 0.1, 0.2, 0.3 in GRM1.

The porosity is found to have a prominent effect on the plasma flow resistance (figure 3*a*). For instance, an increase of *ϕ* = 0.57 to 0.62 and 0.67 for the intermediately disordered LPM (*σ* = 0.5) leads to 24.1% and 39.4% reduction in the pressure drop, respectively. Similarly, the suspension also experiences lower flow resistance: 24.7% and 40.0% reduction, respectively (figure 3*a*). However, the relative apparent viscosity of the suspension remains approximately constant, therefore suggesting a small effect of porosity within the studied range (figure 3*a*). The same trends of pressure drop and relative apparent viscosity against increasing porosity are found for the baseline OPM geometry too (electronic supplementary material, figure S2).

For a given porosity of *ϕ* = 0.57, our simulations in LPM geometries reveal a marginal effect of incremental disorder on the resistance of plasma flow, but an evident effect on that of the suspension flow (figure 3*b*). The relative apparent viscosity increases substantially beyond a critical degree of disorder; *η*_rel_ is 16.4% higher at *σ* = 0.5 compared with its value in the ordered medium (*σ* = 0).

We further investigate the effect of stronger disorder on the relative apparent viscosity of suspension flow with three GRM realizations of distinct topology (but identical porosity and similar throat-width distribution, see figure 1*d*), for which spatial disorder is introduced globally. Different haematocrit levels are examined, where the relative apparent viscosity can rise by nearly 66% as the feeding discharge haematocrit is increased from *H*_*F*_ = 0.1 to 0.3 (figure 3*c*). The viscosity values (e.g. *η*_rel_ ∈ [1.24, 2.05] for GRM1) are in line with recent experimental results for a large-scale porous domain consisting of hexagonal arrays of circular pillars separated by a constant distance of 10 μm [40]. In our case, this distance is represented by the throat width *W*_throat_. For the GRM geometry, *W*_throat_ is not constant, but rather has an exponential distribution (e.g. *W*_throat_ ∈ [2.8, 23.3] μm, with a mean of 8.5 μm and a median of 7.1 μm for GRM1). Through comparison with an established empirical model for blood viscosity *in vitro* [39] (see its formulation in the electronic supplementary material, section S2), our simulated suspension flows at *H*_*F*_ = 0.1, 0.2, 0.3 through the GRM domain are found to have equivalent relative apparent viscosity to a uniform cylindrical tube of 4.2 μm, 4.1 μm, 3.8 μm, respectively (figure 3*c*).

The significantly different effects of porosity and disorder on the relative apparent viscosity are surprising; both decreasing *ϕ* and increasing *σ* are expected to contribute to stronger pore-scale confinement that impedes the transport of RBCs [41] (see definition of the confinement ratio *χ* in §2.1). We believe that this difference in behaviour stems from the sensitivity of local confinement to changes in *ϕ* and *σ*. Changing *ϕ* uniformly modifies all throats (for *ϕ* ∈ [0.57, 0.67]; the mean throat width only varies in the range of ${\bar{W}}_{\mathrm{throat}}\in [5.5,7.5]\;\mu m$). In contrast, a change in *σ* augments the possibility of severe confinement in the system by broadening the *W*_throat_ distribution and giving rise to exceedingly small throats (*W*_throat_ < 3 μm, see figure 1*b*). Note, however, that the impact of occasional severe confinement in some throats when increasing *σ* might be partially compensated by the simultaneously introduced larger throats, leading to an overall minor increment in the relative apparent viscosity.

Theoretically, when the size of a channel approaches that of an RBC, the cell-free layer vanishes due to confinement and the cells are in direct contact with the wall, consequently increasing the relative apparent viscosity of blood in straight tubes as postulated by the well-known Fåhræus–Lindqvist effect [39] (figure 3*c*). However, depending on its severity, such confinement may also contribute to lower relative apparent viscosity by forcing the organization of RBCs into streamlined single-file form, thus excluding excessive cell–cell collisions that would otherwise increase flow resistance [15]. For the disordered geometries studied here, where a tightly interconnected network of pores and throats (rather than isolated channels) are involved, it is expected that both effects exist and compete with each other in determining the relative apparent viscosity of the suspension flow. Further, because each throat has a varying cross-section of contraction and expansion, the spatial organization of RBCs constantly re-arranges without reaching a fully developed profile, which constitutes a mechanism for higher relative apparent viscosity in the porous media than straight tubes of geometrically equivalent diameter (figure 3*c*). These counteracting mechanisms associated with structural alterations sometimes balance each other and the overall viscosity of the suspension remains roughly unchanged under certain circumstances (e.g. figure 3*a*).

### 3.3. RBC traffic dynamically alters the pore-scale flow distribution

**Figure 4.**
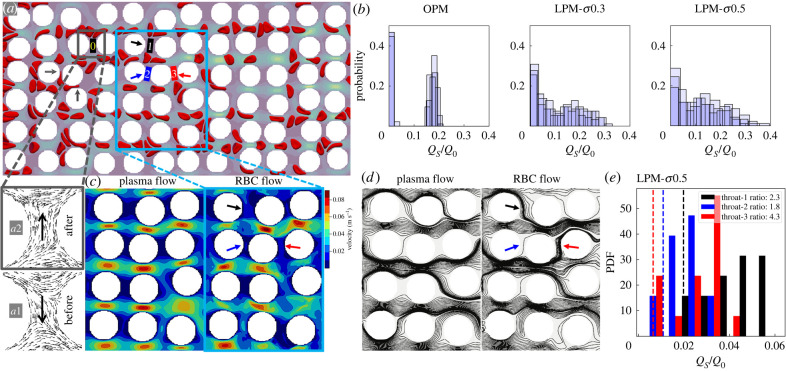
Effect of RBC traffic on local flow patterns. The porosity is *ϕ* = 0.57, and the feeding haematocrit is *H*_*F*_ = 0.2. Flow is from left to right. (*a*) Snapshot of an LPM simulation (*σ* = 0.5). The arrows (dark grey) near the inlet indicate two cell-occlusion events. The small rectangle (dark grey) marks a throat nearby (‘0’) within which flow reversal can be observed before (inset ‘*a*1’) and after (inset ‘*a*2’) the cell-occlusion events. Another rectangle (light blue) marks the central region enlarged in (*c*,*d*) for pore-scale details. (*b*) Flow rate distributions for RBC flows through LPM with increasing levels of disorder *σ* = 0, 0.3, 0.5 (where *σ* = 0 refers to the OPM). The histograms show suspension flow rate magnitudes *Q*_*S*_ evaluated at all throats within the pore space network, and *Q*_0_ = 0.4 μl min^−1^ is the imposed flow rate at the inlet. Three time instants are superimposed to show the temporal fluctuations due to the dynamics of discrete RBCs. (*c*) Instantaneous flow field (plasma-only and suspension) for the same *Q*_0_ = 0.4 μl min^−1^ (*σ* = 0.5). (*d*) Instantaneous streamlines for the same scenarios as in (*c*). The coloured arrows in (*c*,*d*) point to the three throats of interest (‘1’, ‘2’, ‘3’) marked in panel (*a*). (*e*) Quantification of the time-dependent RBC flow through the designated throats of interest as in (*c*,*d*). The histogram for each throat shows the probability of finding the instantaneous flow rate magnitude *Q*_*S*_ normalized by the imposed inflow *Q*_0_; the histograms are obtained by evaluating 15 time instants. The vertical dashed lines represent the corresponding steady and normalized Newtonian plasma flow rates (magnitude) *Q*_*N*_/*Q*_0_. The ratios in the legend indicate the median suspension flow rate divided by the constant plasma flow rate in each throat investigated.

For both the plasma flow and suspension flow (figure 4*a*), a larger degree of structural disorder (i.e. increasing *σ*) is found to promote higher perfusion of the pore space network as indicated by a smaller percentage of throats experiencing negligible flux (compare figure 4*b* with the electronic supplementary material, figure S3A). In both RBC-free and RBC-laden scenarios, the flow distributions evolve from discrete high-/low-flow clusters towards a continuous exponential-like profile as the level of medium disorder increases, similar to earlier findings on Newtonian flows through perturbed cylinder arrays [11]. However, this evolution is noticeably accelerated in the presence of RBCs, i.e. achieving an exponential-like flow distribution at lower level of disorder (figure 4*b*). Given that such exponential distribution is underpinned by less extreme local flow fractions at nodal points (i.e. bifurcations) [11], the RBCs contribute to more robust perfusion of the porous domain, which is in line with a recent report on the role of RBCs in stabilizing blood flow in the capillary bed [42]. Indeed, for strongly disordered geometry where flow channellization (known to be associated with anomalous solute transport in heterogeneous media [43]) prevails in the plasma flow, the presence of RBCs is found to break down some dominant pathways and enhance regional perfusion (see the electronic supplementary material, figure S3B).

Examination of the pore-scale dynamics reveals that the presence of RBCs introduces intermittent and/or permanent occlusion of narrow throats (figure 4*a* and electronic supplementary material, movie S2), featuring trapped RBCs between adjacent cylinders as observed in a recent experimental study [44]. Accompanied by such occlusion events are occasional flow reversals found in throats located in the vicinity (see figure 4*a* insets, ‘*a*1’ and ‘*a*2’). These occlusions may in turn contribute to the diversion of flow (via occlusive pressure feedbacks [45]) to otherwise poorly infused regions (e.g. transversely oriented throats) and lead to more evenly distributed suspension flow in the system (see the enhanced transverse flows in figure 4*c*, right panel) compared with plasma-only flow (figure 4*c*, left panel) under identical inflow conditions. Indeed, the underlying streamline patterns in figure 4*d* suggest that some negligibly perfused throats in the plasma scenario become better perfused in the suspension scenario.

Furthermore, the RBCs impose a temporal signature on the pore-scale flow which is characterized by distinct patterns of flow channellization over time (electronic supplementary material, figure S4). To quantify the temporal variation, three throats of interest (figure 4*e*) are analysed for their instantaneous flow rates over 15 time instants. Wide distributions of flow rates are found, and the time-averaged blood flow at the throats is roughly two to four times as high as in the plasma-only scenario. It is noteworthy that despite the highly dynamic RBC traffic and flow fluctuations at the pore scale, the pressure drop across the entire porous bed is essentially steady (see figure 3 for the narrow error bar ranges).

### 3.4. Symmetry-breaking promotes more homogeneous RBC transport and haematocrit distribution

**Figure 5.**
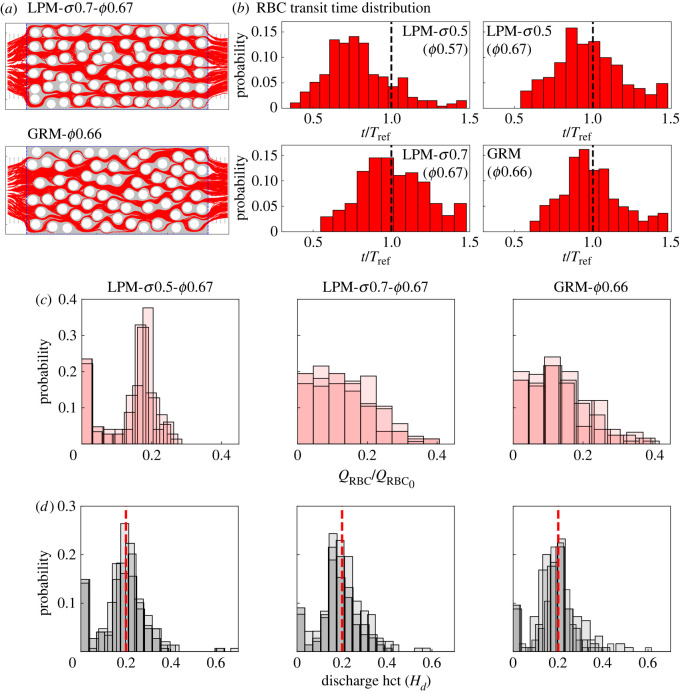
Effect of disorder on RBC perfusion and haematocrit distribution. (*a*) Combined RBC trajectories in simulation over a time period of 45 ms for LPM-*σ*0.7-*ϕ*0.67 and GRM-*ϕ*0.66. (*b*) Distributions of normalized RBC transit time subject to varying levels of porosity and disorder. The transit time is calculated based on each RBC entering and leaving the porous domain between the two vertical dashed lines (blue) in (*a*). The reference transit time *T*_ref_ is defined in appendix A. (*c*) RBC flux distributions corresponding to the high-porosity (*ϕ* = 0.66–0.67) geometries in (*b*). The superimposed histograms for each geometry show the distribution of RBC fluxes evaluated at individual throats throughout the pore space network at three consecutive time intervals. (*d*) Distributions of discharge haematocrits *H*_*d*_ corresponding to the simulated RBC fluxes in (*c*).

RBC trajectories in the porous bed provide information about the spatio-temporal dynamics of the cells (compiled with over 1000 cells, figure 5*a*). Figure 5*b* shows the corresponding distributions of RBC transit time in geometries with different medium disorder. The data reveal that both the distribution shape and median transit time are quantitatively similar in cases investigated here (*ϕ* = 0.67), provided that the porosity remains constant (confirmed for *ϕ* = 0.57 cases in the electronic supplementary material, figure S5A). In other words, the transit time distributions are insensitive to the medium disorder under our studied range of porosity magnitudes. The insensitivity is likely caused by two opposing effects of structural disorder on the motion of RBCs: increasing the tortuosity of existing pathways slows down individual cell transits, but enables more flow pathways by breaking the spatial symmetry and introducing transverse pressure gradients locally. This result suggests that, in a disordered porous medium such as the placental IVS [23], the average residence time of RBCs (associated with oxygenation and solute transport processes) within representative tissue volumes can be independent of the inherent structural heterogeneity if the tissue porosity remains roughly the same.

On the other hand, increasing the porosity from *ϕ* = 0.57 to *ϕ* = 0.67, while keeping the disorder unchanged, leads to an *increase* in median transit time (compare ‘LPM-*σ*0.5-*ϕ*0.57’ and ‘LPM-*σ*0.5-*ϕ*0.67’ in figure 5*b*). This seemingly counterintuitive effect (i.e. slower flow under relaxed confinement) is due to an overall lower interstitial flow speed at higher porosity under the imposition of a *constant* feeding flow rate *Q*_0_ (rather than constant pressure drop Δ*p*). The evidently altered pattern of RBC transit time by a moderate change in the medium porosity suggests a potential mechanism for impaired placental function in pre-eclamptic or diabetic pathologies, where the porous tissues have been found either overly sparser or denser than under normal conditions [46].

Finally, we examine the spatio-temporal RBC distribution in more detail. A visual inspection of the time-lapse RBC perfusion reveals that there are still plenty of throats devoid of RBCs for the intermediate disorder *σ* = 0.5 (electronic supplementary material, movie S4), but for higher degree of a locally perturbed disorder *σ* = 0.7 and the global disorder, the pore space network is more strongly perfused (figure 5*a* and electronic supplementary material, movies S5 and S6). Figure 5*c* shows the RBC flux distributions in the throats of the networks. These data confirm that the locally perturbed ‘LPM-*σ*0.7’ and the globally random ‘GRM’ have comparable RBC distributions while the less disordered ‘LPM-*σ*0.5’ features a large number of throats without RBCs.

The discharge haematocrits *H*_*d*_ of individual throats tend to be close to the feeding discharge haematocrit *H*_*F*_ = 0.2 as the structural disorder becomes sufficiently large in the system (e.g. *σ* ≥ 0.5), featuring a primary peak located around *H*_*d*_ = *H*_*F*_ in the distribution profile (figure 5*d*). This concentrated distribution around *H*_*d*_ = 0.2 is still visible for weakly disordered geometries with *σ* ≤ 0.3, but outweighed by a more dominant peak at zero (see the electronic supplementary material, figure S5B).

Our findings have implications for the optimization of hollow-fibre bundles in extracorporeal membrane oxygenation (ECMO) [47] or microfluidic blood oxygenators in lung assist device (LAD) [26], which adopt designs similar to our studied porous media for oxygen delivery. In both cases, blood is perfused through a porous geometry with uniformly spaced obstacles. Multiple parameters of operation need to be considered for achieving maximal oxygen uptake (e.g. by increasing RBC residence time within the device) while maintaining minimal pressure drop and dampening the temporal fluctuations of oxygenation characteristics induced by flow pulsatility. Our results demonstrate that disorder is another handle to consider for enhancing RBC perfusion, which has not been explored in existing oxygenator designs.

## 4. Conclusion

We have investigated the micro-haemodynamics of cellular blood flow in disordered porous media as simplistic models for extravascular biological tissues, for instance, the intervillous space in the human placenta. Two types of canonical porous media with varying degrees of porosity and disorder were considered: weakly disordered porous media based on a regular square array of non-overlapping circular obstacles, and a random porous medium with intrinsically disordered arrays. We employed simulations combining the lattice-Boltzmann, finite-element and immersed-boundary methods to explore the effects of haematocrit, porosity and structural disorder on the blood rheology, RBC perfusion and haematocrit distribution in the porous media. We also conducted scaled-up experiments using microfluidic droplets to validate the numerical model, where the particle dynamics are found to be qualitatively and quantitatively similar (e.g. flux and velocity distributions). The numerical–experimental framework allows us to examine correlations between the structural characteristics and cellular haemodynamics in canonical disordered porous media, aimed at inferring the structure–function relationship in extravascular biological tissues of high-level heterogeneity and excessive irregularity that remains to be validated using more complicated realistic domains in the future.

The main findings from our study are threefold:

— First, the relative apparent viscosity of cellular blood increases with the structural disorder of the porous media considered herein, but it is largely independent of the studied porosity. These counterintuitive findings are likely caused by competing effects between cell-free layer development (i.e. evolving thickness of depletion layer) and cell re-ordering (i.e. alternating single-file or two/multi-file organization) in narrow channels.
— Second, the presence of RBCs dynamically alters the flow distribution in the investigated porous media. Throats that are only weakly perfused in the absence of RBCs can receive significantly higher flow due to RBCs partially blocking the flow in other, better-perfused throats. Due to the motion of the RBCs, the flow rates in the throats can fluctuate strongly.
— Third, breaking the symmetry of canonical porous media by introducing moderate structural disorder into the system (without causing prohibitive channel occlusions) can promote more homogeneous distribution of RBCs as measured by cell fluxes and discharge haematocrits. While RBCs favour fast lanes through ordered or weakly disordered porous media, a stronger perturbation creates new pathways for the RBCs and lead to more homogeneous haematocrit distribution throughout.

Our reported effects of the porous structure on RBC perfusion may inform more efficient oxygenator designs and our model can be readily applied for simulation as well as optimization of the design. We envision that a multidisciplinary approach, cross-validating simulations and experiments to extract generalized constitutive relationships for RBC flow in complex geometries, will help bridge the gap between microscopic characterization and tissue/organ-level modelling which are both necessary to reveal the relationship between structure and function of biological tissues and organs.

## Data Availability

Data and information supporting this article are provided in the electronic supplementary materials, including one PDF document, six simulation/experiment movies and a ZIP package (SimulationFiles.zip) where simulation parameter files and RBC trajectory data can be found. The source code *HemeLB* for the blood flow simulations in this study is available at https://github.com/hemelb-codes/hemelb. The code version used was f85dac87900e082a6f5fd125a2b6366c94c752e5. The electronic supplementary materials are available online at [48].

## Appendix A

The particle fluxes *Q*_*p*_ (*Q*_RBC_ or *Q*_drop_) and the particle speed *V*_*p*_ (*V*_RBC_ or *V*_drop_) are evaluated at individual throats in the porous domain (L × W × H). Taking the RBC simulations for example, *Q*_RBC_ is defined as

$$Q_{\mathrm{RBC}}=Q_{\mathrm{blood}}H_{d},\;(A\;1)$$

where *Q*_blood_ is the local volumetric flow rate (also denoted as *Q*_*N*_ in Newtonian plasma flow and *Q*_*S*_ in RBC suspension flow) at a given throat and *H*_*d*_ is the discharge haematocrit measured at the same throat. The normalizing particle flux *Q*_RBC0_ is chosen as the overall particle flux fed at the flow inlet; it is defined as

$$Q_{\mathrm{RBC}0}=Q_{0}H_{F},\;(A\;2)$$

where *Q*_0_ and *H*_*F*_ are the flow rate and feeding discharge haematocrit imposed at the inlet, respectively. The normalizing particle velocity *U*_ref_ is chosen as the peak fluid velocity evaluated at the centre of horizontal pores in the baseline OPM geometry, defined as

$$U_{\mathrm{ref}}=\frac{Q_{\mathrm{throat}}}{W_{\mathrm{throat}}H}\;\mathrm{and}\;Q_{\mathrm{throat}}=\frac{Q_{0}}{N},\;(A\;3)$$

where the throat width *W*_throat_ measures the minimum distance between two neighbouring cylinders and *N* is the number of throats across the transverse direction (perpendicular to flow axis) in the OPM geometry. Note that *U*_ref_ should not be mistaken for *U*_0_, which is the superficial velocity defined as

$$U_{0}=\frac{Q_{0}}{WH}.\;(A\;4)$$

*U*_0_ is adopted as spatially averaged advection speed to estimate the reference transit time *T*_ref_ for particles to travel across the whole domain length, defined as

$$T_{\mathrm{ref}}=\frac{L}{U_{0}}.\;(A\;5)$$
